# Life expectancy declines in Russia during the COVID-19 pandemic in 2020

**DOI:** 10.1093/ije/dyac055

**Published:** 2022-03-28

**Authors:** José Manuel Aburto, Jonas Schöley, Ilya Kashnitsky, Ridhi Kashyap

**Affiliations:** Leverhulme Centre for Demographic Science and Department of Sociology, University of Oxford, Oxford, UK; Nuffield College, Oxford, UK; Interdisciplinary Centre on Population Dynamics, University of Southern Denmark, Odense, Denmark; Max Planck Institute for Demographic Research, Rostock, Germany; Interdisciplinary Centre on Population Dynamics, University of Southern Denmark, Odense, Denmark; Max Planck Institute for Demographic Research, Rostock, Germany; Interdisciplinary Centre on Population Dynamics, University of Southern Denmark, Odense, Denmark; Leverhulme Centre for Demographic Science and Department of Sociology, University of Oxford, Oxford, UK; Nuffield College, Oxford, UK

Our recent study of 29 countries, which was published in *IJE*, highlights the devastating impact of the COVID-19 pandemic on mortality across high-income countries.^1^ Period life expectancy witnessed declines in 2020 on a scale that is unprecedented in recent history. With the recent availability of data from Russia,^2^ we show that Russia is no exception (Figure 1). Data from death registrations indicate that life expectancy between 2019 and 2020 in Russia fell by -1.68 (95% CI: -1.72, -1.63) years among males (from 68.41 to 66.73 years) and -1.80 (95% CI: -1.85, -1.76) years for females (from 78.44 to 76.64 years). Comparisons of these losses with those among the 29 countries, as we previously reported,^1^ reveal that life expectancy losses among females were the largest in Russia, and losses among males stand only behind the USA (2.2 years) and Lithuania (1.7 years).

**Figure 1:**
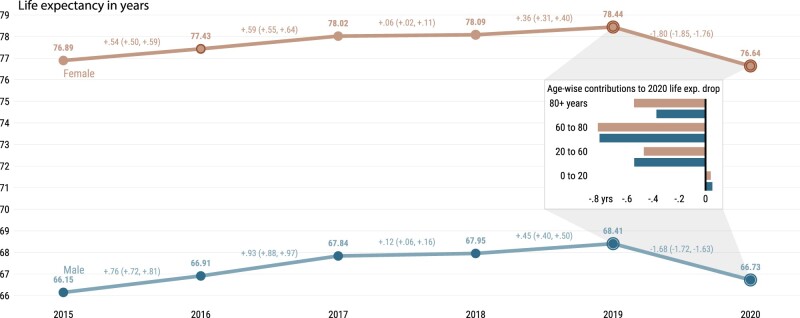
Life expectancy at birth by sex for Russia from 2015 to 2020. The inset shows the age-specific contributions to the change in life expectancy from 2019 to 2020 by sex; 95% prediction intervals via Poisson sampling of age-specific death counts^18^

Two features of the pandemic’s impact on Russian mortality stand out. First, similar to what we found for the USA, increased mortality in working, mid-life ages (20 to 60) accounted for 26% of the total life expectancy decline for women and 33% for men. Since at least 1960, Russia has been the country with the lowest male life expectancy and highest lifespan inequality in Europe, due to a continued burden of mid-life mortality.^3^ Failure to reduce the prevalence of cardiovascular diseases combined with high levels of alcohol consumption and violence led to these patterns.^4^ However, healthier behaviours, better health care and socioeconomic development have led to sizeable improvements in mortality in recent years preceding the COVID-19 pandemic.^4^ In this context of significant progress, the more than one million estimated excess deaths by the end of 2021 [https://github.com/dkobak/excess-mortality],^5^^,^^6^ and the life expectancy declines triggered by excess mortality in mid-life and older-age groups, highlight the catastrophic toll of the pandemic in Russia. To put this into an international perspective, Russia’s level of excess deaths is among the highest across European countries.^5^^,^^7^

Second, sex differences in mortality observed in Russia are striking (Figure 2). Although in most of the 29 countries, we found life expectancy losses to be larger among males than females,^1^ in Russia females experienced larger losses. A potential explanation for the Russian pattern may lie in the heightened exposure to COVID-19 infection experienced by health care workers aged between 20 and 60 during the pandemic, who are predominantly women,^8^ but a deeper assessment of these sex differences is needed.

**Figure 2:**
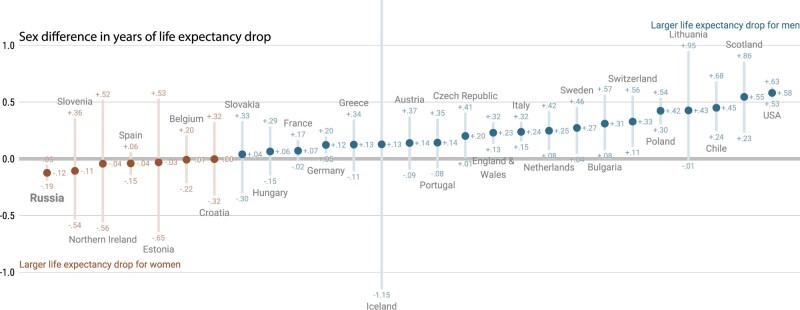
Sex differences in the life-expectancy change from 2019 to 2020 across countries experiencing life expectancy losses; 95% prediction intervals via Poisson sampling of age-specific death counts

Life expectancy losses in Russia in 2020 reveal that they were on a scale comparable to those observed in another similarly large and heterogeneous country, the USA. Russia’s decline in life expectancy may even be larger in magnitude relative to the USA when compared with the projected life expectancy in 2020,^9^ particularly as Russia’s recent progress in life expectancy improvements has been significant compared with the stalls in improvements in the USA. The lack of a streamlined national response, coupled with persistent regional disparities in the implementation and uptake of non-pharmaceutical interventions,^10^^,^^11^ and significant ethno-racial inequalities, are likely to have been factors underlying the significant mortality impacts in both countries.^6^^,^^12^ In Russia, adherence to non-pharmaceutical interventions has been weak and uneven across regions, and distrust in government may have further affected adherence substantially over the course of the pandemic.^13^^,^^14^ The outlook for life expectancy in 2021 is pessimistic as belated efforts to increase vaccination uptake in response to the summer spread of the ‘delta’ variant were subsequently loosened in preparation for parliamentary elections in September 2021, coupled with a strong reluctance to be vaccinated.^10^ National figures are important and informative, but they conceal variation at the sub-national level and by socioeconomic and ethno-racial groups. Evidence from other large and heterogeneous countries, such as Brazil and Mexico,^15^^,^^16^ suggests that life expectancy losses may be more severe in certain regions and larger for disadvantaged groups.^12^ Similarly, the spread of COVID-19 and associated mortality varied between regions in Russia.^7^ Further disaggregated analyses will be needed to understand the evolution of the pandemic and its toll on mortality outcomes, as well as the impact of the more than one million excess deaths up to December 2021.^5^ Moreover, continued poor levels of vaccine uptake in Russia and among Russian-speaking communities across other post-soviet states, in the Baltic countries and Eastern Europe,^17^ indicate that the mortality impacts of the pandemic in these countries in 2021 may yet be worse than in 2020.

## Ethics approval

This research project does not require ethics approval as it uses only macro data that are freely available online.

## Data availability

The replication files for this paper include customized functionality written in the R statistical programming language. The code, and all harmonized input and output data pertaining to our analysis, are hosted on GitHub [https://github.com/oxforddemsci/ex2020].

## Author contributions

Conceptualization: J.M.A., J.S., I.K., R.K. Data curation: J.M.A., J.S., I.K., R.K. Formal analysis: J.M.A., J.S., I.K., R.K. Methodology: J.M.A., J.S., I.K., R.K. Software: J.M.A., J.S., I.K. Visualization: J.S. Project administration: J.M.A., J.S., I.K., R.K. Supervision: J.M.A., J.S., I.K., R.K. Writing—original draft: J.M.A. Writing—review and editing: J.M.A., J.S., I.K., R.K.

## Funding

British Academy’s Newton International Fellowship grant NIFBA19/190679 (J.M.A., R.K.); ROCKWOOL Foundation’s Excess Deaths grant (J.M.A., J.S., I.K.); Leverhulme Trust Large Centre Grant (J.M.A., R.K.).

## Conflict of interest

None declared.
